# A Waveform-Independent Measure of Recurrent Neural Activity

**DOI:** 10.3389/fninf.2022.800116

**Published:** 2022-03-07

**Authors:** Immo Weber, Carina Renate Oehrn

**Affiliations:** ^1^Department of Neurology, Philipps-University Marburg, Marburg, Germany; ^2^Center for Mind, Brain and Behavior (CMBB), Philipps-University Marburg, Marburg, Germany

**Keywords:** oscillations, waveform, recurrence analysis, nonlinear time series analysis, wavelet, rhythmic neural activity

## Abstract

Rhythmic neural activity, so-called oscillations, plays a key role in neural information transmission, processing, and storage. Neural oscillations in distinct frequency bands are central to physiological brain function, and alterations thereof have been associated with several neurological and psychiatric disorders. The most common methods to analyze neural oscillations, e.g., short-time Fourier transform or wavelet analysis, assume that measured neural activity is composed of a series of symmetric prototypical waveforms, e.g., sinusoids. However, usually, the models generating the signal, including waveform shapes of experimentally measured neural activity are unknown. Decomposing asymmetric waveforms of nonlinear origin using these classic methods may result in spurious harmonics visible in the estimated frequency spectra. Here, we introduce a new method for capturing rhythmic brain activity based on recurrences of similar states in phase-space. This method allows for a time-resolved estimation of amplitude fluctuations of recurrent activity irrespective of or specific to waveform shapes. The algorithm is derived from the well-established field of recurrence analysis, which, in comparison to Fourier-based analysis, is still very uncommon in neuroscience. In this paper, we show its advantages and limitations in comparison to short-time Fourier transform and wavelet convolution using periodic signals of different waveform shapes. Furthermore, we demonstrate its application using experimental data, i.e., intracranial and noninvasive electrophysiological recordings from the human motor cortex of one epilepsy patient and one healthy adult, respectively.

## Introduction

During the last two decades, neural oscillations have gained increasing attention as a fundamental mechanism of neural communication (Buehlmann and Deco, 2010; Palmigiano et al., 2017). Neural oscillations are defined as temporally recurring patterns of neuronal activity, also referred to as periodic and rhythmic activity. Oscillations mainly represent synchronized input to neural ensembles consisting of thousands of cells (Buzsáki and Draguhn, 2004). The spatial specificity of recorded activity mainly depends on the measurement device used, with, e.g., surface electrocorticography covering a much broader scale than, e.g., invasive local field potential recordings (LFPs). Classically, oscillatory activity in the human brain is subdivided into five frequency bands: delta (<4 Hz), theta (4–8 Hz), alpha (8–13 Hz), beta (13–30 Hz), and gamma (>30 Hz) (Buzsáki and Draguhn, 2004). A wide range of physiological processes in the animal and human brain is associated with fluctuations of oscillations in distinct frequency bands, e.g., such as deep sleep (delta oscillations, Amzica and Steriade, 1998), long-term memory, and inhibitory top-down control (theta oscillations, Oehrn et al., 2018), attention and local inhibition (alpha oscillations, Bollimunta et al., 2011), and motor control (beta oscillations, Engel and Fries, 2010). Furthermore, alterations in distinct frequency bands occur in neurological and psychiatric diseases, e.g., changes in beta band activity during Parkinson’s disease (Kühn et al., 2006; de Hemptinne et al., 2013; Little and Brown, 2014) and theta activity during essential tremor (Schnitzler et al., 2009; Pedrosa et al., 2012). Nowadays, neuroscientists commonly use wavelet analysis for the quantification of oscillatory activity (van Vugt et al., 2007; Hramov et al., 2015) and Fourier-based analysis tools, such as multitapering or short-time Fourier transformation (van Drongelen, 2018). While being computational efficient, these methods have certain limitations that one needs to consider during interpretation. Classical Fourier and wavelet analysis usually implicitly assume that the analyzed signal is a superposition, i.e., summation of stationary sinusoidal or wavelet-shaped components. However, Fourier transformation of a non-periodic or non-sinusoidal signal may be difficult to interpret. While it is theoretically possible to deconstruct any non-periodic signal into a series of infinite sinusoidals using the Fourier transform, one has to be careful not to overinterpret frequency components, which arise due to the decomposition of a non-periodic non-sinusoidal signal into periodic sinusoidals (Gebber et al., 1999; Lozano-Soldevilla et al., 2016). Thus, the question arises which frequency components do, indeed, carry meaningful information, and which are redundant or even artificial.

The rationale behind using Fourier-based methods is the basic assumption that most electrophysiologically recorded data, e.g., from electroencephalography (EEG) or LFPs, represent the summed activity of large neuronal populations (Franaszczuk and Blinowska, 1985; Buzsáki and Draguhn, 2004). However, despite few attempts at data-driven modeling of specific waveforms (Lewis et al., 2012; Sherman et al., 2016), most often, the signal-generating mechanisms and models and the prototypical waveform shapes of neuronal activity are unknown (Cole and Voytek, 2017). In recent years, waveform shapes have gained increasing interest in the neuroscientific community [for reviews, see Jones (2016) and Cole and Voytek (2017)]. Several studies revealed stereotypical variants of classic frequency bands, which deviated from the sinusoidal waveform shape, e.g., the sensorimotor “mu rhythm,” which is a variation of an alpha wave (Tiihonen et al., 1989; Arroyo et al., 1993; Muthukumaraswamy et al., 2004; Debnath et al., 2019) or motor cortical beta activity with a saw tooth shape (Cole et al., 2017). This non-sinusoidal rhythmic activity is functionally relevant. In Parkinson’s disease, asymmetric beta waves have been associated with the pathological state and shown to become more symmetric with successful treatment, i.e., deep brain stimulation (Cole et al., 2017). While waveform shape is increasingly recognized to carry meaningful physiological information, there is still a lack of tools, which specifically quantify non-sinusoidal activity. While recently, algorithms to characterize waveform shapes have been proposed, methods to incorporate non-sinusoidal activity into frequency analysis are still lacking (Cole et al., 2017; Escobar Sanabria et al., 2017; Pullon et al., 2019). Using classic approaches like Fourier analysis on asymmetric signals leads to the generation of harmonics in the respective spectra, which can be falsely interpreted as meaningful physiological or pathological activity. This is particularly true for measures of coupling, e.g., phase-amplitude coupling where non-sinusoidal signals may lead to spurious results (Lozano-Soldevilla et al., 2016; Yeh et al., 2016).

Here, we introduce a parsimonious way to analyze rhythmic activity that is not based on assumptions regarding waveform shapes. In this approach, we quantify recurrence periods and amplitudes of similar dynamic states based on the established framework of recurrence analysis (Webber and Marwan, 2015). Similar to amplitude or power in Fourier or wavelet analysis, recurrence amplitudes quantify the energy content of the analyzed time series as a function of their (recurrence) frequency. We derive a new algorithm for the estimation of a time-resolved recurrence amplitude spectrum and demonstrate its advantages and limitations in comparison to classic approaches, in particular in regard to non-sinusoidal signals. For this purpose, we use artificial data with known ground truth, as well as real intracranial as well as noninvasive brain recordings from the human motor cortex of one epilepsy patient and one healthy adult, respectively.

## Materials and Methods

### Basic Definitions

For the remainder of this paper, let x_t_ be the realizations of stochastic variables X_t_ at time t, generating a stochastic process *X.* Normal case letters indicate scalar valued observations, while bold letters indicate d-dimensional vector-valued states. A state is defined as a collection of past mostly independent or temporally uncorrelated variables X_t–Δt,_ which are sufficient to predict the present observation X_t_. States can be reconstructed using Takens’ delay-embedding theorem (Takens, 1981) by time shifting the scalar time series *X* (d-1) times by a factor τ = Δt.


(1) $${\text{x}}_{\text{t}}^{{\text{d}}_{\text{x}}}={\left[{\text{x}}_{\text{t}-\left({\text{d}}_{\text{x}}-1\right)\tau },{\text{x}}_{\text{t}-\left({\text{d}}_{\text{x}}-2\right)\tau },\ldots {\text{x}}_{\text{t}-\tau },{\text{x}}_{\text{t}}\right]}^{\text{T}},$$

with *T* indicating the transpose of the vector. The dimension d represents the minimum number of degrees of freedom necessary to sufficiently describe the process *X.*

The collection of all realized states **x**_t_ is defined as the state space of process *X* (Kantz and Schreiber, 2005). For example, a perturbed frictionless pendulum creates a closed trajectory in a two-dimensional phase-space spanned by the variables position and velocity.

Assuming the process *X* to be Markovian, i.e., stochastic with finite memory, the dimension d and the delay τ can be reconstructed from univariate time series using Ragwitz criterion (Ragwitz and Kantz, 2002) or a combination of the false nearest neighbor algorithm (Hegger and Kantz, 1999) and the auto-mutual information (Fraser and Swinney, 1986) (for details, see Supplementary Methods).

### Quantification of Recurrent States

In the following, we will derive an algorithm to estimate the average time-dependent recurrence amplitudes per recurrence frequency of dynamical states. The aim is to derive an estimator to quantify energy distribution of electrophysiological signals as a function of time and frequency similar to short-time Fourier transform (STFT) but based on the concept of recurrent states in phase-space. The idea is that a method based on phase-space observables should be specifically able to quantify high-order oscillations, which are made possible by the combination of uncorrelated succeeding time points to state variables. Thus, this approach might be less redundant in comparison to classic approaches. For this, we will first explain estimation of recurrence periods and recurrence probabilities, which will then be followed by the concept of recurrence amplitudes. Finally, the average recurrence amplitude is calculated by combining the concepts of recurrence probabilities and recurrence amplitudes. Temporal resolution will be introduced by estimating average recurrence amplitudes by using a sliding window.

### Frequency Estimation: The Recurrence Period

A state **x**_t+Δt_ is defined to be recurrent after Δt time steps, if it is within a neighborhood U_ε_ of X_t_ with radius ε (Eckmann et al., 1987):


(2) $${\text{x}}_{t+\Delta t}^{{\text{d}}_{\text{x}}}\in {\text{U}}_{\varepsilon }\left({\text{x}}_{\text{t}}^{{\text{d}}_{\text{x}}}\right)$$

For infinitely small neighborhoods, i.e., ε→ 0, **x**_t+Δt_ is periodic with period Δt, if Δt is the same for all t (Little et al., 2007). All recurrences of an arbitrarily high dimensional phase-space may be represented by calculating a two-dimensional binary recurrence matrix (Marwan et al., 2007):


(3) $${\text{M}}_{t,t+\Delta t}=\Theta \left(\varepsilon -||{\text{x}}_{\text{t}}-x_{t+\Delta t}||\right)$$

**FIGURE 1:**
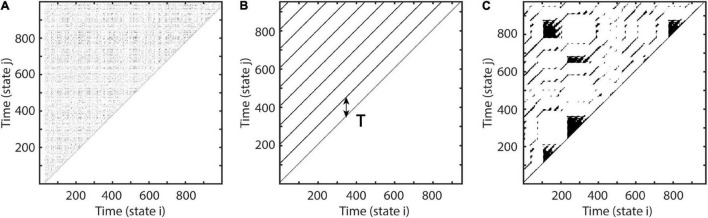
Examples of recurrence plots. At each time i, each black dot indicates a spatial recurrence at time j. Parallel lines indicate periodic activity. Recurrence plots show characteristic patterns, depending on the system’s qualitative dynamics: **(A)** Random white noise. **(B)** Sinusoid with a period of 100 samples. **(C)** A recurrence plot of a classic deterministic nonlinear system, i.e., the Lorenz system (Lorenz, 1963).

Θ is the Heaviside step function and ||.|| is the Euclidean distance norm:


(4) $$||{\text{x}}_{\text{t}}-{\text{x}}_{t+\Delta t}||=\sqrt{\sum_{i=1}^{{\text{d}}_{\text{x}}}{\left({\text{x}}_{i,t}-{\text{x}}_{i,t+\Delta t}\right)}^{2}},$$

where xi is the ith component of phase-space vectors. If (ε-||**x**_t_-**x**_t+Δt_||) is negative Θ is 0 else 1. This recurrence matrix M can be graphically represented by a recurrence plot, where ones are represented by black dots, i.e., recurrences of time i at time j (Figure 1).

**FIGURE 2:**
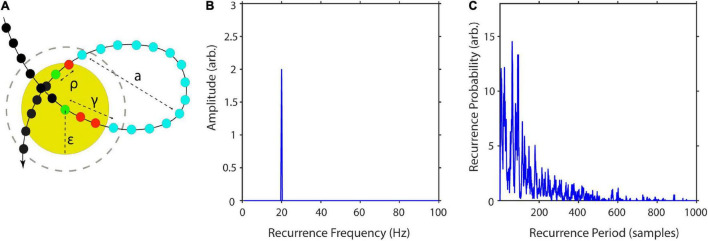
Estimation of recurrence periods. **(A)** A closed local trajectory in phase-space. A reference point (green) is tracked in time until it reenters its spatial neighborhood (yellow circle) with radius ε. In this example, the recurrence period is equal to 14, as it takes 14 samples (blue) until the trajectory reenters the neighborhood. The recurrence amplitude a is estimated as the maximum distance of the recurrence trajectory. Increasing the neighborhood by a multiple of the state-to-state distance (gray dashed circle) reduces the recurrence period by a multiple of two samples. **(B)** Example estimation of recurrence amplitudes using the maximum norm. Recurrence amplitude of 5 s of a 20-Hz periodic signal with an amplitude of two. **(C)** Example estimation of recurrence probabilities for a non-periodic but highly recurrent deterministic system, i.e., the classic Lorenz system (Lorenz, 1963). The plot shows the recurrence probabilities of each period T.

Depending on the system’s local dynamics, the recurrence plot depicts different motifs. Parallel diagonal lines indicate periodicity and determinism, while vertical lines appear due to laminar, i.e., unchanging behavior. White corners arise because of slow drifts or non-stationarity, and isolated dots most often indicate stochastic behavior (Eckmann et al., 1987; Marwan et al., 2007). The recurrence period T of any closest temporal neighbor **x**_t+Δt_ of **x**_t_ within a spatial neighborhood U_ε_ may be estimated as the difference (Little et al., 2007; Ngamga et al., 2007):


(5) T=(t+Δt-ρ)-(t+γ),

where γ is the difference in samples between **x**_t_ and **x**_t_, first leaving U_ε_, and ρ is the sample difference between **x**_t_, reentering U_ε_ and **x**_t+Δt_ (Figure 2A). This is equivalent to estimating the number of vertically aligned, adjacent zeros in the recurrence matrix or the sample length of vertical white lines in the recurrence plot for segments greater than one (see Figure 1B). Excluding consecutive recurrence points (i.e., with period one) has been suggested by Gao (1999) as some of them represent tangential motion instead of the dynamics of the system. Such recurrence periods have been labeled as second type to distinguish them from the original Poincaré recurrence periods (Poincaré, 1890). Subsequently, T is estimated this way for each state.

### Recurrence Probability

By estimating the recurrence period T for all phase-space vectors, it is possible to calculate a histogram R (T), where the bin number is equal to the longest recurrence period T_max_. The recurrence probability may thus be estimated by normalizing R(T) by the total number of recurrence periods (Figure 2C; Little et al., 2007). As noisy experimental data may lead to a high number of short-period recurrences, it is useful to calculate P(T) for a predefined range of T (T_min_–T_max_):


(6) $$\text{P}\left(\text{T}\right)=\frac{\text{R}\left(\text{T}\right)}{\sum_{\text{i}={\text{T}}_{\text{min}}}^{{\text{T}}_{\text{max}}}\text{R}\left(\text{i}\right)},\text{T}={\text{T}}_{\text{min}}\ldots {\text{T}}_{\text{max}}$$

### Amplitude Estimation

As can be seen in Figure 2A, recurrent systems form closed or nearly closed trajectories in phase-space. Here, the energy is contained in the phase-space volume of each recurrent state. In reference to our example, the phase-space portrait would increase in size, if the pendulum would be moved with a greater amplitude. Thus, a reasonable approximation of the amplitude of each period would be to estimate its maximum diameter in phase-space.


(7) $$\bar{a}\left(\text{T}\right)=\sum_{k=1}^{\text{q}}\text{max}||{\text{x}}_{\text{i}}-{\text{x}}_{\text{j}}||\cdot {\text{q}}^{-1},\forall \text{i},\text{j}\in \left\{1\ldots \text{n}\right\}$$

with q being the number of recurrences per T, i.e., how often a recurrence of duration T has occurred, i and j being the sample indices of the kth recurrence, and n being the number of samples per recurrence (see Figure 2A).

This may be repeated for each recurrent trajectory per recurrence period T and subsequently averaged (Figure 2B). The average over all recurrences per T is taken here to avoid a bias toward rare recurrences with large maximum diameters, which might occur due to measurement noise. A similar approach has been previously described for the detection of synchronization for non-phase-coherent and non-stationary data by Romano et al. (2005). Finally, we weighted each estimated mean amplitude by its recurrence probability to get an average for each recurrence frequency bin. This is equivalent to estimating the expected value of the amplitude for every recurrence frequency:


(8) $$\text{A}\left(\text{T}\right)=\text{P}\left(\text{T}\right)\cdot \bar{a}\left(\text{T}\right)$$

This is done to negate the effect of noise on recurrence amplitude estimation. Rare occurrences of high-amplitude recurrences in the vicinity of the “true” recurrence frequency will thus get diminished if their recurrency probability is lower than the “true” recurrence frequency (for a comparison with unweighted recurrence amplitudes, see Section “Comparison between recurrence estimation, Fourier transform and wavelet transform”).

### Time-Resolved Recurrence Amplitude

In neuroscientific research, it is often of interest to analyze spectral activity changes relative to some intervention, e.g., some stimulus or response. For this purpose, methods like STFT or wavelet transform estimate power spectral density as a function of time (Hramov et al., 2015). Similarly, the recurrence amplitude spectrum may be estimated for n short overlapping time windows w_n_ of definite length:


(9) $$\text{P}\left(\text{T},{\text{w}}_{\text{n}}\right)=\frac{\text{R}\left(\text{T},{\text{w}}_{\text{n}}\right)}{\sum_{\text{i}={\text{T}}_{\text{min}}}^{{\text{T}}_{\text{max}}}\text{R}\left(\text{i},{\text{w}}_{\text{n}}\right)},\text{T}={\text{T}}_{\text{min}}\ldots {\text{T}}_{\text{max}}$$

The length of each time window determines the maximum resolvable recurrence period and should thus be chosen with respect to the minimum frequency of interest.

### Influence of Neighborhood ε on Recurrence Estimation

The recurrence probability is an estimate of the probability of a recurrence occurring after T time steps. The estimation of P(T) is dependent on ε. For experimental data, a too small choice of ε would result in many empty neighborhoods and thus in a high degree of statistical errors due to measurement noise. If ε is chosen too large, recurrences are not local anymore, and recurrence periods are underestimated. The recurrence period gets approximately underestimated by two samples for every increase of ε by a multiple q of the state-to-state distance || **x**_t+1_-**x**_t_|| (e.g., see the gray dashed circle in Figure 2A). Thus, the number recurrence times of period T in R gets added to bins of lower periods:


(10) $$\begin{array}{c} {\text{R}}_{\text{err}}\left(T,\varepsilon_{\text{err}}\right)\approx R\left({\text{T}}_{\text{err}}\right)\\ {\text{T}}_{\text{err}}\left(q,\varepsilon_{\text{err}}\right)\approx \text{T}-2q, \end{array}$$

with


(11) $$\varepsilon_{\mathrm{err}}\approx \varepsilon +\text{q}\cdot ||{\text{x}}_{t+1}-{\text{x}}_{\text{t}}||,$$

**FIGURE 3:**
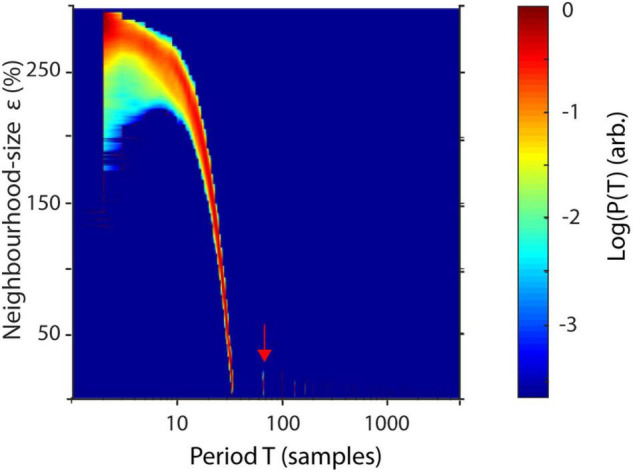
Spatially resolved recurrence probability. The plot shows recurrence probabilities P(T) of 50 s of a 3-Hz signal (+8% STD white noise) as a function of periods T = 1–5,000 samples and increasing neighborhood sizes ε = 1–300. ε is represented in % of the standard deviation of the raw time series. The arrow indicates the noise floor.

To better understand the influence of the parameter ε on recurrences, P(T) may be estimated over a wide range of neighborhood-sizes, resulting in a spatially resolved recurrence period spectrum (SREPS). In the SREPS, one would expect to find three regions of interest, depending on ε. For very small ε, the SREPS is governed by statistical errors and a uniform distribution across all T. For very high ε, the distribution of P(T) is heavily shifted to small T with only few state vectors, leaving and reentering any neighborhood, with the extreme case of a neighborhood size fully engulfing the phase-space. If the signal contains any oscillatory activity, slowly shifting, but continuous “spectral” peaks occur in the intermediate range of ε. As stated above, the best estimate of the recurrence period may thus be found at the crossing of the continuous spectral peaks and the noise regime for low ε (Figure 3).

### Comparison Between Recurrence Estimation, Fourier Transform, and Wavelet Transform

**FIGURE 4:**
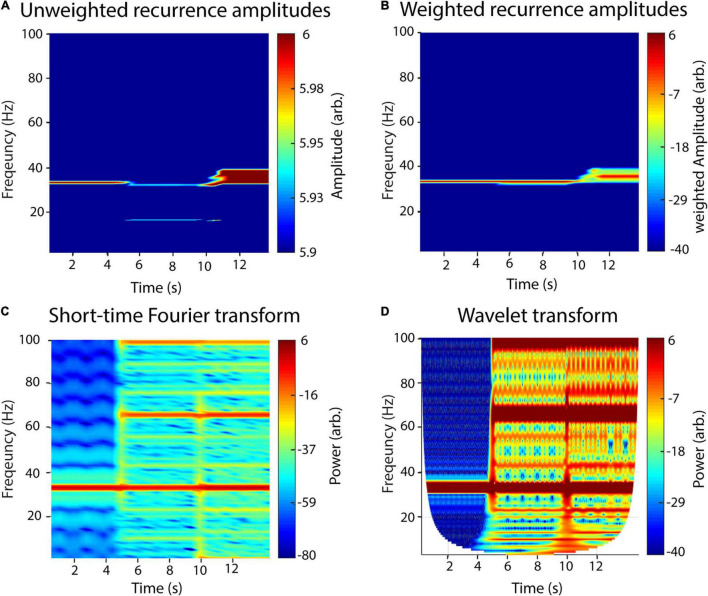
A recurrence amplitude spectrum. The recurrence amplitude spectrum of a compound signal of three 33-Hz oscillatory signals of different shapes is shown. The first 5 s is composed of a sine wave, the next 5 s of a sawtooth wave, and the last 5 s of a rectangle wave. **(A)** Raw unweighted amplitudes. **(B)** Raw amplitudes were weighted with their respective probability densities. **(C)** Short-time Fourier transform of the same signal. Window length was set to 1 s with a 50% overlap. Note the spurious harmonics for the nonlinear waveform shapes, i.e., rectangle (5–10 s) and sawtooth shape (10–15 s). **(D)** Wavelet transform of the signal. Wavelet width was set to 30. Note the spurious harmonics for the nonlinear waveform shapes, i.e., rectangle (5–10 s) and sawtooth shape (10–15 s).

In Figure 4, we demonstrate the raw (Figure 4A) and a weighted recurrence amplitude spectrum (Figure 4B) using an artificial signal of three signals of different concatenated waveform shapes, each with a length of 5 s, a frequency of 33 Hz and an amplitude of two: (1) a sinusoid, (2) a sawtooth wave and (3) a rectangle wave. While the frequency resolution succeedingly decreases for the three waveform types using the raw amplitude spectrum, it stays nearly the same for the weighted spectrum. However, the amplitude of the rectangle curve is slightly under- and its frequency slightly overestimated. Using the weighted amplitude spectrum increased the frequency resolution for the non-sinusoidal signals. For comparison with classic approaches, we analyzed the same signal with STFT (Figure 4C) and wavelet analysis (Figure 4D). For the recurrence amplitude spectrum and the short-time Fourier spectrum, we used 50% overlapping windows of 600-ms lengths. For wavelet analysis, we used 30 cycles in order to approximate the window length for the other methods (1 s). For STFT and wavelet analysis, spurious harmonics can be seen for the non-sinusoidal waveform shapes in addition to the true frequency at 33 Hz. In contrast, the weighted recurrence amplitude spectrum shows only one spectral peak at a relatively narrow frequency band.

**FIGURE 5:**
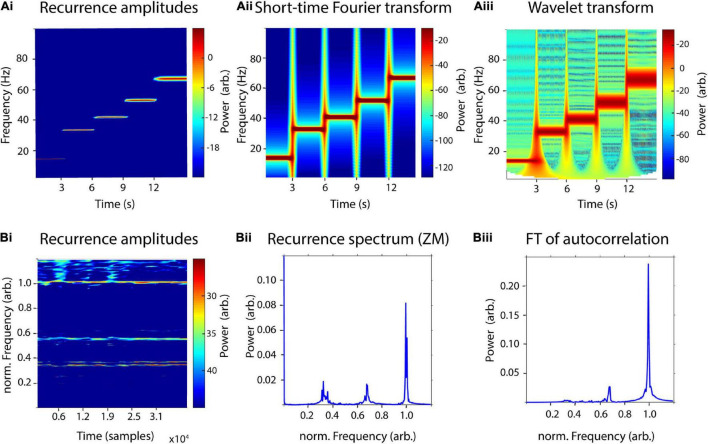
Sensitivity to time-frequency transitions and multiple high-order frequencies. **(A)** Comparison of a time-resolved recurrence amplitude spectrum **(Ai)** with short-time Fourier transform **(Aii)** and wavelet transform **(Aiii)** using a concatenated signal of five 3-s segments with increasing frequencies (14, 33, 41, 52, and 67 Hz). **(B)** Comparison of a time-resolved recurrence amplitude spectrum **(Bi)** with a time-independent recurrence spectrum proposed by Zbilut and Marwan (2008) **(Bii)** and the Fourier transform of the autocorrelation function **(Biii)** of 40,000 samples of the Belousov–Zhabotinsky chemical reaction. The period-3 orbit between the frequencies of 0.3 and 0.4 can only be detected in panels **(Bi,Bii)**. Frequencies were normalized to the fundamental period of the system at 125 samples.

In order to test the sensitivity of the recurrence amplitude spectrum to time-frequency transitions, we concatenated five 3 s signals of increasing frequencies (14, 33, 41, 52, and 67 Hz) and compared results with short-time Fourier transform and wavelet transform (Figure 5A). We used the same parameters as before, i.e., a window length of 1,000 samples with a 50% overlap for recurrence amplitudes and STFT and 30 cycles for the wavelet transform. The five different frequencies were reliably detected with all three methods. However, the sharp transitions of segments led to broadband power increases in the STFT and wavelet transform, which could not be observed in the recurrence amplitude spectrum. For both, wavelet and recurrence spectrum higher frequencies led to a worse frequency resolution, although the differences between frequencies for the latter were much smaller.

Zbilut and Marwan (2008) have previously proposed a similar but time-independent, recurrence-based amplitude spectrum. However, the main difference to our proposed estimator is that Zbilut and Marwan combine recurrence analysis with Fourier transform. For this, they apply the Fourier transform to the generalized autocovariance function derived from recurrence quantification to estimate a recurrence-based power spectrum. They demonstrate their method using a time series of the nonlinear Belousov–Zhabotinsky chemical reaction. Here, we used 40,000 samples of the same dataset to compare our proposed method to Zbilut’s and Marwan’s estimator as well as to a Fourier transform of the standard autocorrelation of the time series (Figure 5B). According to Lathrop and Kostelich, the system’s attractor exhibits a main periodic orbit with a period of approximately 125 samples, as well as a period-2 and period-3 orbit at about 250 and 375 samples, respectively. Similar to Zbilut and Marwan, we normalized frequencies to the period-1 orbit (125 samples) and used an embedding dimension *d* = 3 and a delay τ = 124. We used a window size of 2,000 samples with a 50% overlap for the time-resolved recurrence amplitude spectrum. The generalized autocorrelation function was estimated using the nta_recurrenceplot.m function of the NoLiTiA-Toolbox (Weber and Oehrn, 2021). The period-1 and period-2 could be reliably detected with all three methods. However, the period-2 orbit was estimated at a slightly lower frequency for the recurrence amplitude spectrum in comparison to both other methods (0.35 vs. 0.67). The period-3 orbit at approximately 0.32 could only be shown with Zbilut’s and Marwan’s method and our proposed estimator. Interestingly, the period-3 orbit exhibits a double peak in the spectrum of both methods, which has not been shown in Zbilut’s and Marwan’s original study in which they used only 1,000 samples of the time series.

### Influence of Measurement Noise

**FIGURE 6:**
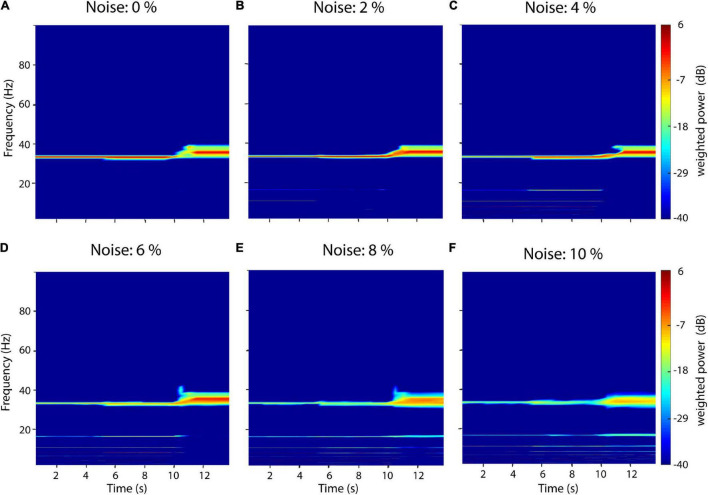
Influence of white noise on recurrence amplitude estimation. Increasing white noise levels between 0 and 10% of the raw signal’s amplitude was added to a compound signal composed of three waveform shapes: 33-Hz sinusoid (0–5 s), a 33 Hz sawtooth wave (5–10 s), and a 33 Hz rectangle wave (10–15 s). **(A)** 0% white noise. **(B)** 2% white noise. **(C)** 4% white noise. **(D)** 6% white noise. **(E)** 8% white noise. **(F)** 10% white noise. Subharmonics appear with increasing noise levels.

Measurement noise in electrophysiological recordings is typically in the range between 2 and 7% of physiological amplitudes (Ryynänen et al., 2004). To test the influence of measurement noise on recurrence amplitude estimation we thus added increasing uniform white noise levels from 0 to 10% of the signal’s amplitude to the same test signal described in Section “Comparison between recurrence estimation, Fourier transform and wavelet transform” (Figure 6). Neighborhood-size ε was set *ad hoc* at 10% STD. The added noise leads to the occurrence of subharmonics for all three signal types. For the sinusoid and sawtooth wave, spurious subharmonics at 16.4 and 11 Hz first occur at 2% noise (Figure 6B). With increasing noise levels, increasingly more subharmonics can be detected in the spectrum (Figures 6C,D). Finally, starting at 8 Hz subharmonics at 16.4 and 11 Hz can also be detected for the rectangle wave (Figures 6E,F). In contrast to Fourier-based methods, white noise does not increase the baseline power but, instead, leads to the detection of subharmonics. This is because immediate recurrences of the original signal might get randomly missed with increasing noise levels but get still detected after n periods.

### Waveform-Specific Filter

**FIGURE 7:**
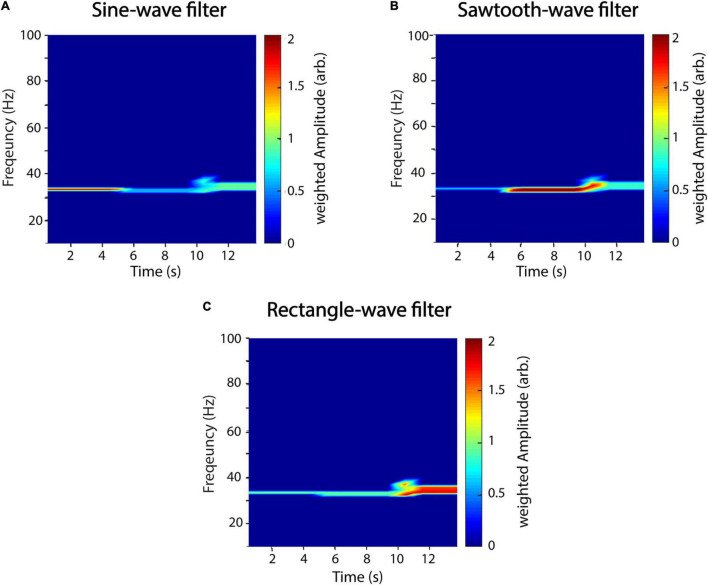
A recurrence amplitude spectrum with templates. The recurrence amplitude spectrum of a compound signal of three 33-Hz oscillatory signals of different shapes and a waveform-dependent gain is shown. The first 5 s is composed of a sine wave, the next 5 s of a sawtooth wave, and the last 5 s of a rectangle wave. Shown are the raw unweighted amplitudes. **(A)** Five cycles of a sine wave were used as a shape template. **(B)** Five cycles of a sawtooth wave were used as a shape template. **(C)** Five cycles of a rectangle wave were used as a shape template. α was always set to 5. Window length was set to 1 s with a 50% overlap.

While the proposed estimator is independent of waveform, it is possible to tailor it to distinct waveform shapes. For this, we introduce a gain factor to the amplitude estimation step, which is simply the Pearson correlation of the waveform shape of each recurrence and a scaled template waveform ζ.


(12) $$\bar{a}\left(\text{T}\right)=\sum_{k=1}^{\text{q}}\text{max}||{\text{x}}_{\text{i}}-{\text{x}}_{\text{j}}||\cdot {\text{G}}^{\alpha }\cdot {\text{q}}^{-1},\forall \text{i},\text{j}\in \left\{1\ldots \text{n}\right\}$$

with α being chosen arbitrary and G being the gain factor:


(13) $$\text{G}\left(\text{k}\right)=\text{max}\left(\frac{\text{COV}\left(\text{x},\zeta_{\text{m}}\right)}{\sigma_{\text{x}}\sigma_{\zeta^{\text{m}}}}\right),\text{m}=\zeta_{\text{m}}\ldots \zeta_{m+T},$$

with cov being the covariance, σ being the variance, and ζ_m_ being the template waveform from sample m to m + T. For perfectly matching waveforms, the gain is unity, and, thus, the amplitude estimation is unaffected (Figure 7). In Figure 7, we used the same artificial signal as in Figure 4 and analyzed it using waveform templates of a sinusoid (Figure 7A), sawtooth (Figure 7B), and rectangle shape (Figure 7C), respectively. The exponent α determines the strength of the gain factor and thus how much specific waveform shapes are filtered. In this example, it was set to five. Figure 7 demonstrates that using specific waveforms as filters successfully attenuates power of time series with non-matching shapes. However, attenuation is not perfect, and the specificity of the filtering seems to be dependent on the specific shape template as can be seen, e.g., for the sine-wave filter (Figure 7A), where residual power can be detected for the sawtooth and, to a higher degree, for the rectangle wave. Thus, while sine-wave and sawtooth-wave filters attenuate each other rather well, the effect is smaller for the rectangle-wave filter. A possible explanation might be that the rectangle-wave shares features with both, the sine-wave and the sawtooth-wave, in that it has a similar symmetry as the former and rather sharp transitions like the latter.

### Implementation and Workflow

The proposed method is implemented in the nta_windrecfreq.m function of the NoLiTiA-Toolbox (Weber and Oehrn, 2021). NoLiTiA is a free, open-source MATLAB Toolbox with methods from dynamical systems theory, information theory, and recurrence analysis. Most functions require two input arguments: (1) time series data and (2) a configuration structure containing parameters for estimation. The most important parameters for nta_windrecfreq.m are summarized in Table 1.

**TABLE 1:** Main input parameters for nta_windrecfreq.m.

Parameter	Description	Input type	Default
cfg.tau	Embedding delay τ for phase-space reconstruction	integer number	0 (automatic optimization using auto-mutual information)
cfg.dim	Embedding dimension d for phase-space reconstruction	Integer number	0 (automatic optimization using false nearest neighbors algorithm)
cfg.en	Neighborhood-size ε in % STD of the time series	Integer number	5 (5%)
cfg.metric	Distance norm used to estimate distances in phase-space	String (“euclidean”/“maximum”)	maximum (maximum norm)
cfg.window	Length of sliding window w in ms	integer number	1/10 of data length
cfg.fs	Sampling frequency fs of time series	integer number	−
cfg.amplitudes	Estimate amplitudes (yes/no)	integer number (0/1)	1 (yes)
cfg.minmaxRecPD	Minimum and maximum recurrence periods to consider	vector (start end)	[0 0] (consider all possible periods)
cfg.db	Export amplitudes in decibel (yes/no)	integer number (0/1)	1 (yes)
cfg.outp	Export amplitudes either as magnitude or power	string (“amp”/“pow”)	“amp” (magnitude)

**FIGURE 8:**
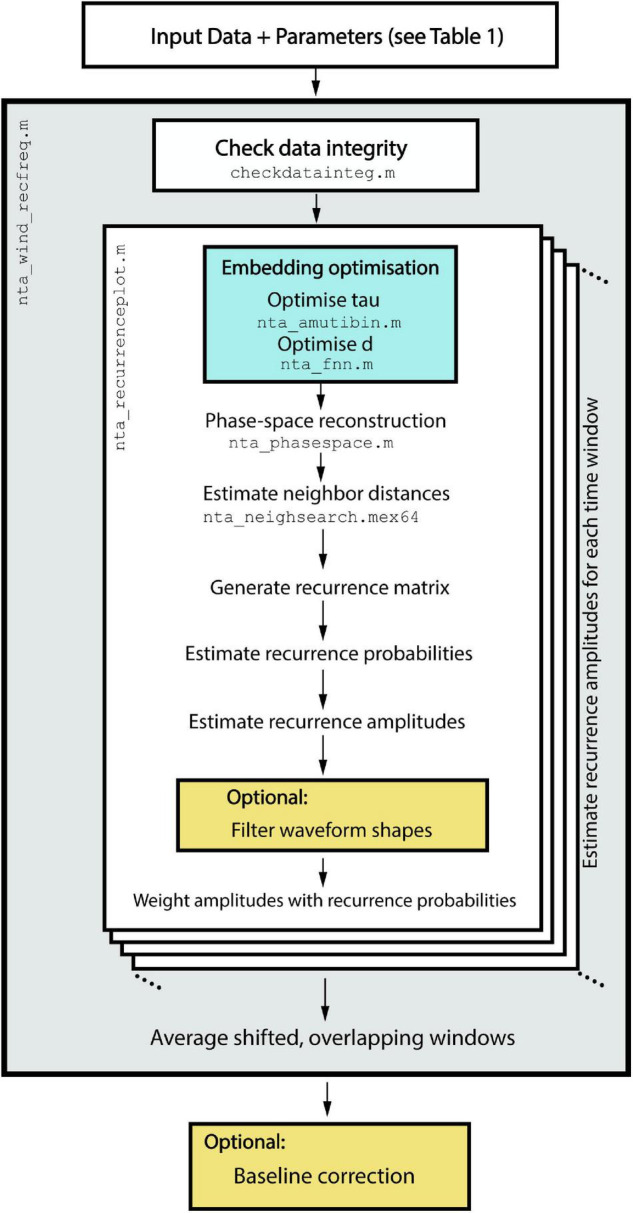
Workflow of the implemented algorithms in nta_wind_recfreq.m. After time series data and the configuration structure are passed to nta_recfreq.m, it iteratively calls nta_recurrenceplot.m to estimate recurrence amplitudes for each shifted time window.

*STD, standard deviation; ms, milliseconds.*

nta_windrecfreq.m is a wrapper function, which iteratively calls nta_recurrenceplot.m for each time window and subsequently averages the overlapping segments. nta_recurrenceplot.m is the main function, which embeds the time series in phase-space, estimates neighbor distances, generates the recurrence matrices, estimates recurrence probabilities, and finally estimates amplitudes, which get weighted by their respective probabilities (see Figure 8). The most important helper functions include nta_amutibin.m and nta_fnn.m, which both optimize embedding delay and dimension, respectively, nta_phasespace.m, which embeds the time series and nta_neighsearch.mex64, which estimates distances of all neighbors in phase-space. The latter was implemented as a mex-file to reduce computation time.

**FIGURE 9:**
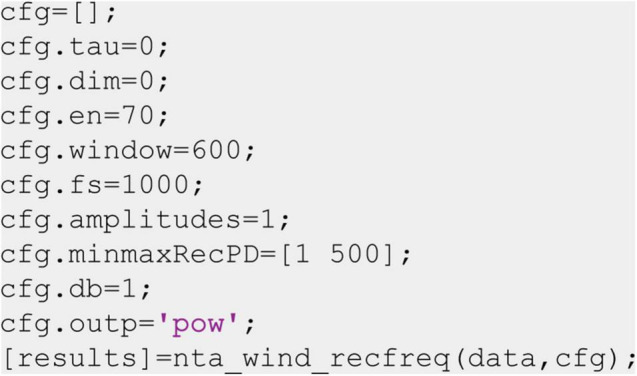
An example script to call nta_wind_recfreq.m. For description of parameters, see Table 1.

An example script to call nta_windrecfreq.m is depicted in Figure 9. Setting cfg.tau and cfg.dim to zero optimizes both embedding parameters automatically. At a sampling rate of cfg.fs = 1,000 Hz, a window length of cfg.window = 600 samples allow for a minimum resolvable recurrence frequency of 1.67 Hz. cfg.window should be chosen according to the minimum frequency intended to analyze. However, increasing the window length also reduces the temporal resolution of recurrence amplitude estimation. Thus, cfg.window is always a compromise between minimum resolvable recurrence frequency and temporal resolution. The parameter cfg.en defines the neighborhood size in % of the STD of the input time series. Since the neighborhood size should ideally cover the measurement noise, a good starting point for cfg.en is a range between 50 and 100% of the time series’ STD. There is no general consensus on how to define ε for experimental data and, as such, data should only be interpreted in the light of transparent reporting. An extensive documentation on all parameters and all functions and subfunctions can be found in the documentation of the NoLiTiA-toolbox^1^ or by the standard MATLAB help function.

### Example Application

**FIGURE 10:**
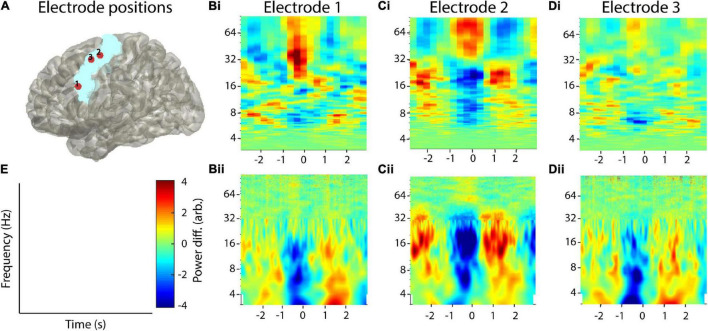
Example application on real invasive EEG data. **(A)** Selected electrodes in the motor area of one patient with epilepsy. The blue area indicates the precentral gyrus located with the AAL atlas in FieldTrip. **(Bi–Di)** Recurrence analysis of three example electrodes. Plots have been convolved with a 5 × 5 smoothing kernel for visualization purposes. **(Bii–Dii)** Corresponding combined multitaper-wavelet analysis of the same electrodes. All power values were normalized with respect to the total power of the analyzed time window. **(E)** Colorbar and axis labels for Subfigures **(B–D)**.

While the recurrence amplitude spectrum might be estimated for all types of oscillatory activity, it is particularly suited for the analysis of electrophysiological neural activity. Thus, we briefly demonstrate its application using intracranial brain recordings from the motor cortex of one epilepsy patient. The patient received a 48 electrode electrocorticography (ECoG) grid for diagnostic purposes. Electrodes were localized by co-registration of preoperative MRI and postoperative CT scans using the FieldTrip toolbox (Oostenveld et al., 2011; Figure 10A). As oscillatory activity in the motor cortex has been well characterized in previous studies, we selected three electrodes located on the precentral gyrus, i.e., the motor cortex (two medial electrodes in the proximity of the hand area and one lateral electrode). Anatomical selection was based on the AAL atlas implemented in FieldTrip (Tzourio-Mazoyer et al., 2002; Figure 10A). We recorded electrophysiological data by means of the Neurofax-system of Nihon Kohden (Nihon Kohden, Rosbach, Germany) at a sampling rate of 1,000 Hz. The patient participated in a study where he was asked to press a button on a standard computer keyboard (*n* = 96 trials). To minimize edge effects occurring after convolution with a wavelet kernel, we segmented the data into relatively large time intervals of 4 s before until 4 s after the response onset and discarded 1 s on each end of the segment after wavelet convolution. We removed line-noise, visually rejected artifacts, and performed trend correction. We performed a response-locked data analysis, i.e., neural data are analyzed time locked to the patient’s button press. To this end, we used the nta_wind_recfreq.m function implemented in the NoLiTiA-Toolbox and compared results to classic time-frequency analysis, i.e., wavelet convolution, in order to analyze power in different frequency bands. We used window lengths of 600 samples (=0.6 s, parameter: cfg.window) and a 50% overlap for the time-resolved estimation of recurrence amplitudes (Figures 10Bi–Di, colorbar and axis labels shown in Figure 10E). Phase-space parameters, i.e., dimension d and delay τ were optimized for each time window using false nearest-neighborhood algorithm and auto-mutual information, respectively (parameters: cfg.dim and cfg.tau were both set to zero). Neighborhood size was chosen *ad hoc* at 70% of the standard deviation of each time series (parameter: cfg.en). For comparison with classic methods, we used a combined wavelet (for frequencies, 3–30 Hz) and multitaper approach (for frequencies above 30 Hz; Figures 10Bii–Dii, colorbar and axis labels shown in Figure 10E), as suggested for better frequency resolution and frequently adopted in the literature (van Vugt et al., 2007). To this end, we convolved the data with a continuous complex Morlet wavelet with seven cycles. From the wavelet-transformed signal, we extracted power values between 3 and 30 Hz in 1 Hz steps (lower frequencies are difficult to estimate using wavelet convolution in short time windows). For the multi-taper analysis, we used eight tapers and a sliding 600 ms time window centered at 1-ms steps. Both spectra were normalized, using the complete time period as the baseline.

**FIGURE 11:**
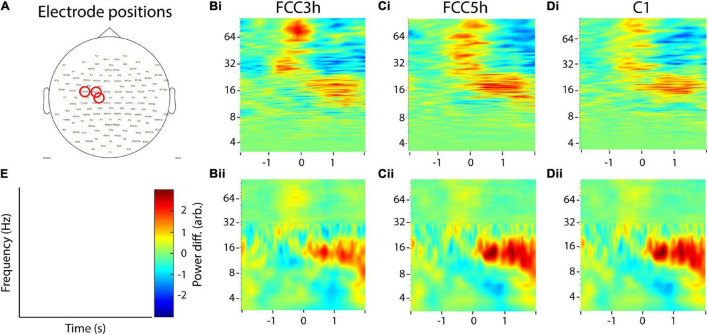
Example application on noninvasive EEG data. **(A)** Selected electrodes in the motor area of one healthy adult. **(Bi–Di)** Recurrence analysis of three example electrodes. **(Bii–Dii)** Corresponding combined multitaper-wavelet analysis of the same electrodes. All power values were normalized with respect to the total power of the analyzed time window. **(E)** Colorbar and axis labels for Subfigures **(B–D)**.

To complement this analysis, we also analyzed the data set of one healthy adult who was recorded with noninvasive EEG. Similar to the first data set, the proband was asked to press a button on a standard computer keyboard (*n* = 190 trials). For analysis, we used three electrodes in the left hemispheric primary motor area (FCC3h, FCC5h, C1, Figure 11). All analysis parameters were the same as for the invasive EEG electrodes.

Both study protocols were approved by the medical Ethics Committee Marburg and conducted in accordance with the latest version of the Declaration of Helsinki.

## Results

### Invasive Recording

Using combined multitaper-wavelet analysis, we found a desynchronization around the button press (∼−1–0 ms), ranging across frequency bands delta to beta (∼3–32 Hz) in all three electrodes (Figures 10Bii–Dii). After the motor response (∼1 s post-response), we observed an enhancement in activity in this broad spectrum approximately 1 s after the response. This so-called rebound activity occurred in a broad frequency range from delta to low gamma with it, being most prominent in the beta band of electrode two. This beta rebound is a well-described phenomenon in the motor cortex during motor tasks (Crone et al., 1998; Pfurtscheller and Da Lopes Silva, 1999; Jurkiewicz et al., 2006; Miller et al., 2007). Recurrence analysis showed a more specific beta desynchronization and rebound for electrode two, which was also more narrowband. In contrast to wavelet analysis, we additionally found a broad gamma activation during the button press, which was most prominently followed by a gamma desynchronization in electrode two but also visible in electrodes one and three to a lesser degree. In contrast, recurrence analysis did hardly reveal any changes in the theta band. Taken together, recurrence analysis was more sensitive for broad band high frequency activity that was not detected by wavelet convolution.

### Noninvasive Recording

Analysis of the noninvasive recordings provided similar results as for the invasive recordings. In all three electrodes, a pronounced beta rebound between 0 and 2 s post button-press could be observed, both with the combined multitaper-wavelet approach and recurrence analysis. However, the effect was stronger using the multitaper-wavelet approach. For both methods, the beta desynchronization around the button press was much weaker in comparison to the invasive recordings. Again, for the recurrence analysis, a stronger gamma activation can be observed around the button press (Figure 11).

### Statistical Analysis

**FIGURE 12:**
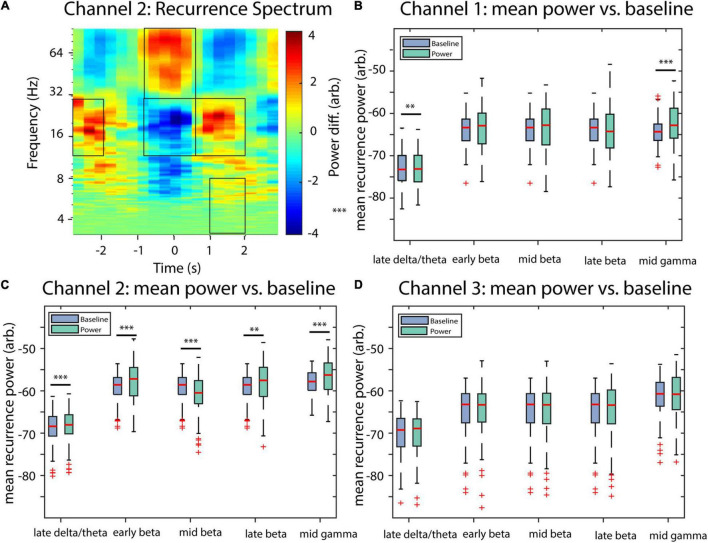
Statistical comparisons of recurrence amplitudes with baseline activity of invasive recordings. **(A)** An example recurrence amplitude plot, indicating the time-frequency intervals extracted for statistical analysis. **(B)** Statistical comparisons for Channel 1 (two-sided paired Student’s *T*-test, Bonferroni corrected). **(C)** Statistical comparisons for Channel 2. **(D)** Statistical comparisons for Channel 3. Asterisks indicate significant differences (^**^p < 0.01, ^***^p < 0.001).

Results of the recurrence analysis of the invasive recordings were statistically tested using a similar approach as described, e.g., in de Hemptinne et al. (2015) for cross-frequency coupling, which, similar to time-resolved recurrence amplitudes, is also a two-dimensional measure (i.e., time vs. frequency for former and frequency of phase vs. frequency of amplitude for latter measure). For both measures, the aim is to statistically validate hotspots of their respective two-dimensional distributions. For this, we averaged recurrence amplitudes trial-wise over five different combinations of frequencies and time intervals and compared these averages to their respective baseline activities using a two-sided paired Student’s *T*-test at an alpha level of 5% (Bonferroni corrected). The baseline data consisted of the temporal average of the complete trial, which was subsequently expanded to its original duration. The extracted combinations of frequencies and time intervals include late delta-theta (3–8 Hz, 1 to 2 s), early beta (13–30 Hz, −3 to −2 s), mid beta (13–30 Hz, −0.5 to 0.5 s), late beta (13–30 Hz, 0.5 to 2 s), and mid gamma (30–100 Hz, −0.5 to 0.5 s, Figure 12A). For Channel 1, late delta/theta power and mid gamma power were both larger than the baseline (late delta/theta: *p* = 0.008, mid gamma: *p* < 0.001, Figure 12B). For Channel 2 late delta/theta, early beta, late beta, and mid gamma power were increased, while mid beta power was decreased in comparison to the baseline (late delta/theta: *p* < 0.001, early beta: *p* < 0.001, mid beta: *p* < 0.001, late beta: *p* = 0.0098, mid gamma: *p* < 0.001, Figure 12C). All other comparisons were non-significant (*p* > 0.055; for multitaper-wavelet results, see Supplementary Figure 1).

## Discussion

Here, we introduce a new method to analyze oscillatory activity in neural systems. We validated our approach with synthetic data and demonstrated its application with real experimental invasive EEG data of one epilepsy patient and noninvasive EEG data of one healthy adult. Using artificial data, we show that the power estimates resulting from our method are more frequency specific for non-sinusoidal waveforms and are not associated with spurious harmonics visible in the estimated frequency spectra. However, by applying specific waveform templates, we demonstrated that our method is also able to detect specific shapes if necessary. Using human EEG recordings from the motor cortex of one epilepsy patient and one healthy adult, we showed that recurrence analysis compares to conventional methods, such as wavelet analysis and Fourier transformation, in detecting movement-related oscillatory activity in the motor cortex.

With both methods, we found beta desynchronization in the motor cortex around the button press followed by a beta rebound (Salmelin et al., 1995; Jurkiewicz et al., 2006; Parkes et al., 2006). Both methods indicate that this effect is spatially specific, as it was most prominent in one of the two medial electrodes in proximity of the hand area of the primary motor cortex. This was validated by statistical analysis, which showed that the beta rebound using recurrence analysis was only significantly different from the baseline in Channel 2.

Furthermore, recurrence analysis was more sensitive for detecting broad band gamma activity in Channels one and two, which is thought to represent multi-unit activity rather than narrowband oscillations (Leszczyński et al., 2020). Recurrence estimation was seemingly less sensitive for theta and delta oscillations revealed by wavelet analysis, although statistical differences could still be detected for Channels one and two. In contrast to beta activity in electrode two, theta/delta was more broadband in all three electrodes for wavelet analysis and much more narrowband for recurrence analysis. It is possible that this activity does not represent true oscillatory or recurrent activity but, rather, local non-stationarities or drifts, which can hardly be detected with recurrence analysis. This is because states of local non-stationarities, even if they are oscillatory, do not come sufficiently close to each other after the full period to enter their respective neighborhoods.

Despite their fundamental differences in estimation procedures, it is evident that classic methods (i.e., wavelets and taper) and our proposed approach capture recurrent neural activity, which validates both methodological concepts from different angles. However, discussing the mathematical equivalence of recurrence analysis and classic methods is beyond the scope of this study and should be analyzed in future research.

Classically, neural oscillatory activity is analyzed using methods derived from Fourier transform. Using these methods, time series get decomposed into prototypical waveforms or “wavelets” e.g., sinusoids. While this approach is justified by the understanding that, e.g., EEG activity is a summation or superposition of thousands of synchronously active cells (Buzsáki and Draguhn, 2004), interpretation of spectral estimation may be limited in some cases. This is because the generating models of neural activity and thus the basal waveform shape are most often unknown. Decomposing an asymmetric or nonlinear waveform into a series of sinusoids results in an infinite number of spurious harmonics (as demonstrated in Figures 4C,D), which may be misinterpreted as independent oscillatory activity. Thus, for such nonlinear signals, classic techniques may generate a high degree of redundant information. Note, however, that wavelet analysis is in theory capable of redundantly quantifying non-sinusoidal oscillations by applying special mother wavelets, e.g., like the Daubechies wavelets (Zhang et al., 2016). However, one drawback is that using a specific mother wavelet would still restrict analysis to one specific waveform shape and would also require prior knowledge, while recurrence-based methods may detect unspecified arbitrary shapes. It is also still very uncommon to use any other mother wavelet than the Morlet wavelet for oscillatory analysis, although few studies exist that used other types to detect recurring spiking events in the EEG (Milton, 1994; Grubov et al., 2017). The problem of nonsinusoidal waveform shape has been especially demonstrated for connectivity measures, i.e., cross-frequency coupling (Lozano-Soldevilla et al., 2016; Yeh et al., 2016). Occurrences of nonlinear signals in electrophysiology are increasingly recognized to be commonly present in physiological (Arroyo et al., 1993; Gebber et al., 1999; Muthukumaraswamy et al., 2004; Lozano-Soldevilla et al., 2016) and pathological states (Cole et al., 2017). The physiological meaning of waveform shape, however, is still insufficiently understood (Cole and Voytek, 2017). One prominent example of a nonsinusoidal oscillation is the cortical mu-rhythm, which is composed of an alpha and a beta component, together resulting in an arc-shaped wave. Source reconstruction studies hint at a superposition of alpha and beta oscillations originating from different cortical regions, which are responsible for the generation of the mu-wave (Hari and Salmelin, 1997; van Wijk et al., 2012). Whether or not Mu can, indeed, be redundantly described as a single oscillatory phenomenon may be further investigated using recurrence amplitude estimation in future studies but is beyond the scope of this study.

The approach applied in this study utilizes the concept of recurrences in phase-space. The probability of a state recurrence as a function of its period has been frequently studied (Gilmore, 1993; Romano et al., 2005; Little et al., 2007; Webber and Zbilut, 2007; Zou et al., 2007). Our method builds upon this by also estimating amplitudes, i.e., energy content of a recurrence by estimating the phase-space volume of the recurrence. Additionally, by windowing the estimation procedure, it is also possible to calculate a time-resolved recurrence amplitude spectrum, similar to STFT or wavelet analysis. The major difference to Fourier-based techniques is that time series are not fitted to basal waveforms in a “model-based” kind of way. Instead, it is quantified after what time the system reassumes a previous state, independent of the specific waveform in between these recurrences. Thus, as has been demonstrated in this study, simple asymmetric waveforms can be described more parsimoniously, without any spurious harmonics (Figures 4A,B). The most extreme example of this is a rhythmic idealized Dirac pulse, which has unity power over all frequencies in Fourier space (Beerends, 2006) but can be parsimoniously represented with recurrence-based methods. Thus, another possible application of our proposed method might be analysis of spike train or electromyography data (EMG). The problems regarding the analysis of the latter with Fourier-based methods are widely recognized, which is why EMG data are often additionally preprocessed by, e.g., extracting the Hilbert envelope or taking the absolute value (Myers et al., 2003). These preprocessing steps may, however, lead to spurious results, depending on further analysis (McClelland et al., 2012; Negro et al., 2015). We could also demonstrate that our estimator could reliably detect sharp time-frequency transitions in a similar manner as STFT and wavelet transform (Figure 5A). However, while STFT and wavelet transform exhibited broadband power increases at the transitions between different frequency segments, the recurrence amplitude spectrum lacked these broadband peaks. The explanation for this is the same as the one above, concerning the Dirac pulse, as sharp transitions can only be represented in Fourier space by infinite harmonics, which necessarily result in spurious broadband power increases.

Our proposed estimator is not the first study on a recurrence amplitudes spectrum. Zbilut and Marwan (2008) proposed an estimator, which combines the so-called generalized autocovariance with Fourier transformation. In their study, they could demonstrate superiority of their method in comparison to classic Fourier analysis by showing that only their estimator could detect all main periodic orbits in a nonlinear system. Here, we used the same dataset to compare our method with Zbilut’s and Marwan’s estimator as well as with a classic Fourier-based power spectrum (Figure 5B). We could demonstrate that both our estimator and Zbilut’s and Marwan’s method could detect all three periodic orbits, while classic Fourier analysis detected only two of three frequencies. Thus, our estimator is sensitive to periodic oscillations of nonlinear systems with the additional advantage of being time resolved. While an advantage of Zbilut’s and Marwan’s estimator is a much smoother spectrum, a disadvantage might be the possibility that spurious harmonics might still occur, if the estimated generalized autocovariance is discrete or has sharp transitions.

Although neuroscience is among the main fields of application for recurrence-based methods, they are still scarcely applied in comparison to classic Fourier-based approaches to oscillatory analysis. Examples include classification of mild cognitive impairment (Timothy et al., 2017), multiple sclerosis (Carrubba et al., 2019) or emotional states (Khodabakhshi and Saba, 2020). One possible reason for this might be the number of parameters that need to be adjusted. While the algorithm is not parametric, i.e., not model based *per se*, the estimation procedure may be sensitive to several key parameters due to the finiteness of measured data. These most prominently include the neighborhood-size ε and the embedding parameters d and τ. For perfectly periodic recurrences and infinitely precise sampled data, the neighborhood size may be chosen arbitrarily small. However, for experimental data, ε should be ideally chosen to barely engulf most of recurrent states. If the neighborhood size is chosen too large, recurrence periods get underestimated for every multiple of the sampling frequency. If ε is chosen slightly too small, recurrences might be missed. This may result in “harmonics,” i.e., multiples of recurrence periods appearing in the spectrum, as recurrences missed in one period might get detected in the next one (as can be seen in Figures 3, 6). However, as our algorithm weights amplitudes by their probability of occurrences, few missed recurrences do not severely impact the overall spectrum. In the case that ε is chosen much too small, all meaningful recurrences might be missed and the spectrum gets dominated by measurement noise. The choice of ε thus depends on experimental data. However, it is important to note that, for real experimental data, there is no true neighborhood size as neural systems are hardly ever perfectly periodic. For intermediate ranges of ε, recurrence spectra are rather stable and frequencies should only slowly shift (Figure 3). From a practical point of view, it is convenient to approximate the measurement noise as the standard deviation of the electrophysiological signal (Ryynänen et al., 2004). Thus, a good starting point for ε might be at 100% standard deviation, which might be successively reduced if no drastic qualitative changes appear in the spectrum (e.g., subharmonics). Nevertheless, when reporting results, neighborhood and embedding parameters should always be reported for reproducibility. One possible approach to optimize neighborhood size is to estimate the recurrence amplitude spectrum as a function of ε and visually identify the noise regime for small neighborhood sizes (Figure 3). However, as this might be quite computationally demanding, it suffices to estimate the non-time resolved spectrum for a subsample of the data. This is justified if the variance of noise is static over time. The subset should be chosen long enough to cover the longest recurrence period of interest.

Of similar importance is an appropriate embedding of the measured data in phase-space. If the embedding dimension is too low, points, which are far away from each other, might get projected into close proximity. Thus, the time in between might be spuriously characterized as a specific recurrence period. On the other hand, if the embedding dimension is too high, estimation of recurrence periods becomes increasingly computational demanding and neighboring points difficult to detect due to the increasing spaces between points, otherwise known as “curse of dimensionality.” The embedding delay is important for spreading out the phase-space volume. A delay, which is too small, would result in all points laying on the first intersect and thus no closed trajectories to measure. For our algorithm we used the well-established false-nearest neighbors algorithm (Hegger and Kantz, 1999) for the optimization of the embedding dimension and the auto-mutual information for the embedding delay (Fraser and Swinney, 1986). However, other techniques like, e.g., the Ragwitz-algorithm, are also frequently reported to optimize embedding parameters (Ragwitz and Kantz, 2002; Lindner et al., 2011; Weber et al., 2020). By automatically optimizing d and τ, we effectively eliminate these parameters, which makes the estimation procedure much easier to apply. This procedure is well established and implemented in many toolboxes utilizing the concept of phase-space analysis (Lindner et al., 2011; Lizier, 2014; Donges et al., 2015).

Similar to Fourier-based techniques, the window size should be chosen according to the smallest frequency of interest. For example, a window with a length of 1,000 samples at a sampling rate of 1,000 Hz would allow for the detection of precisely one recurrence at 1 Hz. However, to reliably detect recurrences at a given frequency, the window length should be chosen at a multiple of the desired frequency, e.g., three times the period of interest.

One limitation of the recurrence analysis is that it is, by design, not able to decompose a linear superposition of sine waves. The difference between recurrence-based and Fourier-based techniques is that the latter transforms the signal parametrically to the frequency domain, which is orthogonal to the time domain. In contrast, recurrence-based methods track higher order state recurrences within a given time window. Both approaches have distinct advantages and disadvantages. While the Fourier-based methods are able to parametrically decompose a superposition of oscillations, they introduce redundancy if either the rhythmic activity is not perfectly periodic or if it is nonlinear. While they are similar, both approaches differ in what questions they ultimately want to answer. For Fourier-based approaches, one intends to find out which composition of sinusoids forms the signal in question. If the original signal is already an artificial superposition of sinusoids to begin with, Fourier-based methods are optimally suited for analysis. On the other side, recurrence-based methods use a more data-driven approach in that they directly track recurring patterns in the time domain. We thus propose to apply the demonstrated technique complementary in conjunction with classic approaches, e.g., to discern possible spurious harmonics.

In this study, we introduced a new time-resolved technique to measure amplitudes of oscillatory signals in a waveform-independent manner. This method estimates the energy of recurrent activity by measuring distances of closed trajectories in phase-space, which are subsequently weighted by their respective probability densities. Using artificial data, we demonstrated that the measure generates less spurious harmonics due to nonlinear waveform shapes in comparison to classic techniques like Fourier Transform. Furthermore, the analysis of intracranial and noninvasive data indicates that recurrence analysis might be better suited to estimate higher frequency activity than congenital methods, such as wavelet analysis or the Fourier transform. In addition, we showed that recurrence analysis can be used to specifically analyze signals with defined waveform shapes. In summary, the proposed measure might be well suited to complement classic frequency techniques, especially when the analyzed signals are of nonlinear origin.

## Data Availability Statement

The datasets and scripts used for this study can be found at https://rushfiles.one/client/publiclink.aspx?id=I6lhffuqnm. The proposed method is implemented in the function nta_wind_recfreq.m of the NoLiTiA-Toolbox which may be downloaded at https://nolitia.com/download. The full documentation of implemented functions and parameters can be found in the user manual provided with the toolbox. The time series of the Belousov Zhabotinsky chemical reaction can be downloaded at https://complex.umd.edu/data/bzflow2.zip.

## Ethics Statement

The studies involving human participants were reviewed and approved by Ethics Committee of the medical faculty of the Philipps-University Marburg. The patients/participants provided their written informed consent to participate in this study.

## Author Contributions

IW developed and implemented the measures, performed the analyses, and wrote the manuscript. CO performed the measurements, wrote the manuscript, discussed results, and helped supervising the project. All authors contributed to the article and approved the submitted version.

## Conflict of Interest

The authors declare that the research was conducted in the absence of any commercial or financial relationships that could be construed as a potential conflict of interest.

## Publisher’s Note

All claims expressed in this article are solely those of the authors and do not necessarily represent those of their affiliated organizations, or those of the publisher, the editors and the reviewers. Any product that may be evaluated in this article, or claim that may be made by its manufacturer, is not guaranteed or endorsed by the publisher.

## Footnotes

1
https://nolitia.com/download
