# Supplementary material for: A Waveform-Independent Measure of Recurrent Neural Activity
Source: Front Neuroinform. 2022 Mar 7;16:800116. doi: 10.3389/fninf.2022.800116 (PMC8936506; doi:10.3389/fninf.2022.800116)
Supplement: Supplementary file 1 [file Data_Sheet_1.PDF]

Supplementary methods

Ragwitz criterion

Assuming the process *X* to be Markovian i.e. stochastic with finite memory, the dimension d and the delay τ can be reconstructed from univariate time series using Ragwitz criterion (Ragwitz and Kantz 2002). For a given combination of d and τ, neighbours of every state x_t within a spherical neighbourhood U_ε of diameter ε are iterated one time step. The mean of iterated neighbours is the prediction $\hat{\mathbf{x}}_{t+1}$ of $\mathbf{x}_{t+1}$.

$$\hat{\boldsymbol{x}}_{t+1}^{d_x} = \frac{1}{\left|U_\varepsilon(\boldsymbol{x}_t^{d_x})\right|} \sum_{\boldsymbol{x}_{t-\Delta t}^{d_x} \in \boldsymbol{U}_\varepsilon(\boldsymbol{x}_t^{d_x})} \boldsymbol{x}_{t-\Delta t+1}^{d_x} \tag{S1}$$

Where t-Δt indicates that spatial neighbours temporaly precede $\mathbf{x}_t$ and |.| indicates the number of neighbours in U_ε. The combination of d and τ is chosen for which the root mean squared prediction error is minimum:

$$RMSPE = \sqrt{\frac{\sum_{t=1}^{n}\left(\hat{\boldsymbol{x}}_{t+\Delta t}^{d_x} - \boldsymbol{x}_{t+\Delta t}^{d_x}\right)^2}{n}} \tag{S2}$$

where n is the number of states predicted.

False nearest neighbours algorithm

For deterministic systems, the phase-space dimension may be alternatively reconstructed using the false nearest neighbourhood method (FNN, Hegger and Kantz 1999):

$$\mathrm{FNN(d)} = \sum \frac{\theta\left(\frac{\left|\mathrm{x}_{i+1} - \mathrm{n}(\mathrm{x}_i)_{j+1}\right|}{\left\|\mathbf{x}_i^d - \mathbf{n}_{xi}^d\right\|} - \mathrm{Rtol}\right)}{\wedge\ \theta\left(\sqrt{\left(\left\|\mathbf{x}_i^d - \mathbf{n}_{xi}^d\right\|\right)^2 - \left(\left|\mathrm{x}_{i+1} - \mathrm{n}(\mathrm{x}_i)_{j+1}\right|\right)^2} - \mathrm{SD(X)} * \mathrm{Atol}\right)}, \tag{S3}$$

with θ indicating the Heaviside-step function, i being the temporal index of x, j representing the temporal index of n, $n(x_i)$ indicating the next neighbor of x_i, ||..|| indicating the maximum norm, R_{tol} being a distance threshold, A_{tol} being a loneliness threshold and SD indicating the standard deviation. If the embedding dimension is too low, false neighbours of points in phase-space may arise due to projections. The optimal embedding dimension is thus the dimension for which the percentage of false neighbours drops to zero.

Auto-mutual information

The embedding delay may be estimated using the auto-mutual information (AMI, Fraser and Swinney 1986). Here, the shared information between the present and past of a process X is calculated as a function of a delay Δt. The Δt for which AMI drops to zero may be used as τ for the embedding:

$$AMI(\mathrm{X_t}; \mathrm{X_{t-\Delta t}}, \Delta\mathrm{t}) = \mathrm{H}(\mathrm{X_t}) - \mathrm{H}(\mathrm{X_t}|\mathrm{X_{t-\Delta t}}), \quad \text{(S4)}$$

with

$$\mathrm{H(X)} = -\sum \mathrm{p(X = x)} \log_2 \mathrm{p(X = x)} \quad \text{(S5)}$$

and p(X=x) being the probability of X taking on the value of x.

Publication bibliography

Fraser; Swinney (1986): Independent coordinates for strange attractors from mutual information. In *Physical review. A, General physics* 33 (2), pp. 1134–1140. DOI: 10.1103/physreva.33.1134.

Hegger, R.; Kantz, H. (1999): Improved false nearest neighbor method to detect determinism in time series data. In *Physical review. E, Statistical physics, plasmas, fluids, and related interdisciplinary topics* 60 (4 Pt B), pp. 4970–4973. DOI: 10.1103/physreve.60.4970.

Ragwitz, M.; Kantz, H. (2002): Markov models from data by simple nonlinear time series predictors in delay embedding spaces. In *PHYSICAL REVIEW E* 6505 (5), p. 6201.
