# Supplementary material for: A Waveform-Independent Measure of Recurrent Neural Activity
Source: Front Neuroinform. 2022 Mar 7;16:800116. doi: 10.3389/fninf.2022.800116 (PMC8936506; doi:10.3389/fninf.2022.800116)
Supplement: Supplementary file 2 [file Data_Sheet_2.PDF]

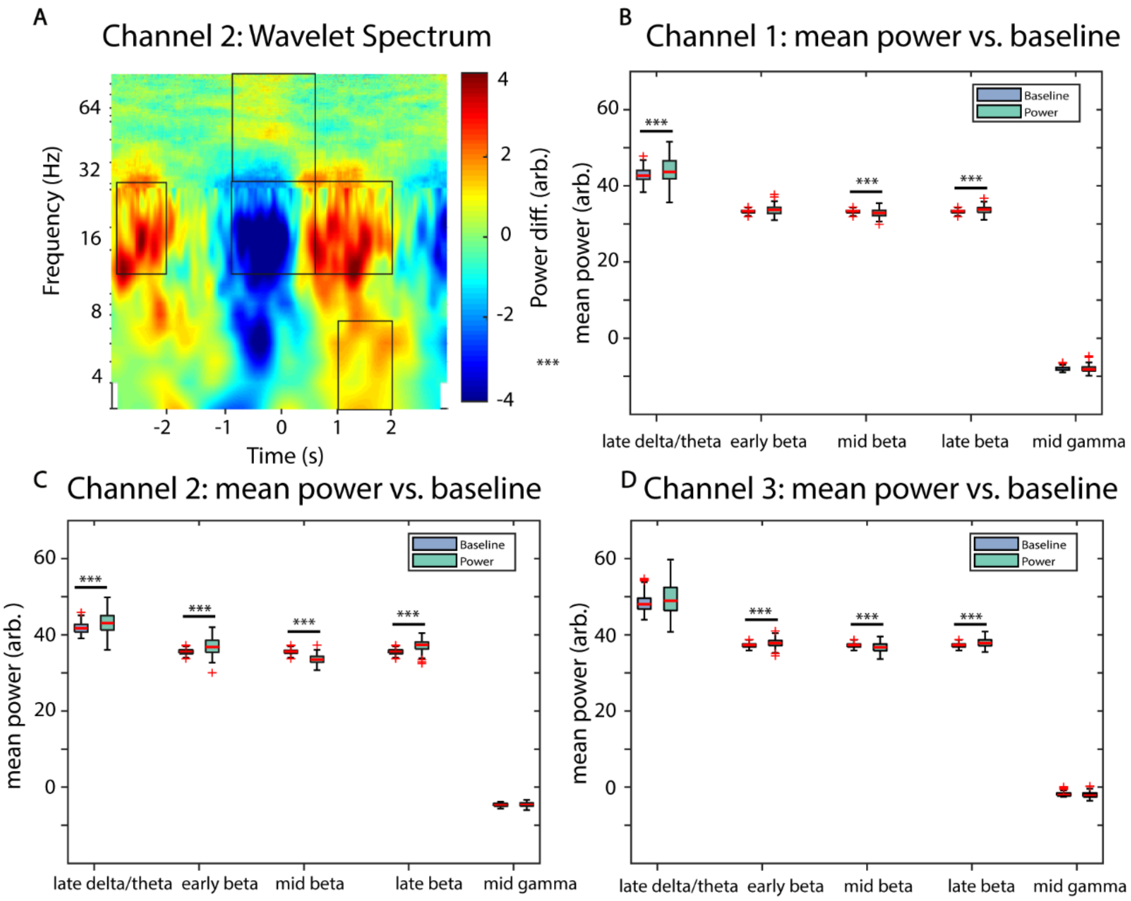

Supplementary Figure S1: Statistical comparisons of combined multitaper-wavelet power with baseline activity. A: Example multitaper-wavelet plot indicating the time-frequency intervals extracted for statistical analysis. B: Statistical comparisons for channel 1 (two-sided paired Student's t-test, Bonferroni corrected). C: Statistical comparisons for channel 2. D: Statistical comparisons for channel 3. Asterisks indicate significant differences (***: $p<0.001$).
